# Association between multi-site atherosclerotic plaques and systemic arteriosclerosis: results from the BEST study (Beijing Vascular Disease Patients Evaluation Study)

**DOI:** 10.1186/s12947-020-00212-3

**Published:** 2020-08-01

**Authors:** Huan Liu, Jinbo Liu, Wei Huang, Hongwei Zhao, Na Zhao, Hongyu Wang

**Affiliations:** 1 Vascular Medicine Center, Peking University Shougang Hospital, NO. 9 Jinyuanzhuang Road, Shijingshan District, Beijing, China; 2 Vascular Health Research Center of Peking University Health Science Center, Beijing, China; 3 Key Laboratory of Molecular Cardiovascular Sciences (Peking University), Ministry of Education, Beijing, China; 4 Peking University Clinical Research Institute, Beijing, China

**Keywords:** Arteriosclerosis, Atherosclerosis, Atherosclerotic plaque, Carotid femoral artery pulse wave velocity, CF-PWV

## Abstract

**Background:**

Arteriosclerosis can be reflected in various aspect of the artery, including atherosclerotic plaque formation or stiffening on the arterial wall. Both arteriosclerosis and atherosclerosis are important and closely associated with cardiovascular disease (CVD). The aim of the study was to evaluate the association between systemic arteriosclerosis and multi-site atherosclerotic plaques.

**Methods:**

The study was designed as an observational cross-sectional study. A total of 1178 participants (mean age 67.4 years; 52.2% male) enrolled into the observational study from 2010 to 2017. Systemic arteriosclerosis was assessed by carotid femoral artery pulse wave velocity (CF-PWV) and multi-site atherosclerotic plaques (MAP, > = 2 of the below sites) were reflected in the carotid or subclavian artery, abdominal aorta and lower extremities arteries using ultrasound equipment. The associations were assessed by multivariable logistic regression.

**Results:**

The prevalence of CF-PWV > 12 m/s and MAP were 40.2% and 74.4%. Atherosclerotic plaques in 3 sites were more common in male compared with that in female (48.9% versus 36.9%, *p* < 0.05). All CVD factors were worse in participants with MAP than that with <=1 site. Participants with CF-PWV > 12 m/s corresponded to a mean 82% probability of MAP with age and sex-adjusted. Patients with peripheral artery disease showed the highest odds ratio (OR) (3.88) for MAP, followed by smoking (2.485), CF-PWV > 12 m/s (2.25), dyslipidemia (1.89), male (1.84), stroke (1.64), hypoglycemic agents (1.56) and age (1.09) (all *p* < 0.001).

**Conclusions:**

MAP was highly prevalent in this cohort, with male showing a higher prevalence than female. Higher systemic arteriosclerosis was independently associated with MAP, which indicating the supplementary value of arteriosclerosis for the earlier identification and intervention on MAP.

**Trial registration:**

Clinical Trial, URL: http://www.clinicaltrials.gov. Unique identifier: NCT02569268.

## Introduction

Arteriosclerosis (arterial stiffening) assessed by carotid femoral artery pulse wave velocity (CF-PWV) and atherosclerosis (plaque formation) reflected in the carotid using ultrasound equipment, have been considered as independent predicting factors of cardiovascular disease (CVD), and are recommended by guidelines on risk prediction [1–5]. A meta-analysis indicated that 27.22% of Chinese people aged 30–79 years were with carotid atherosclerosis in 2010, equivalent to 207.73 million affected individuals. With demographic ageing, the number of people affected by carotid atherosclerosis will increase to 267.25 million by 2020 [6]. Carotid atherosclerosis alone has such a high disease burden that it is necessary to assess atherosclerosis in other vascular beds, such as subclavian artery, abdominal aorta and lower extremities arteries. Given the systemic nature of atherosclerosis, a multi-site detection on the others vessels will provide a more comprehensive evaluation on the atherosclerotic plaque burden.

The comprehensive evaluation on vascular health include not only the structure (atherosclerotic plaque formation) but also the structure (vascular wall elasticity or arteriosclerosis). However, few studies have evaluated that whether higher arteriosclerosis is more vulnerable to the formation of multi-site atherosclerotic plaques (MAP). Therefore, the present study was to evaluate the association between systemic arteriosclerosis and multi-site atherosclerotic plaques in the carotid or subclavian artery, the abdominal aorta and lower extremities arteries using noninvasive ultrasound imaging techniques in an in-hospital cohort. By evaluating multiple vascular beds, we aim to improve our understanding on the atherosclerotic distribution and explore the potential association between MAP and CF-PW.

## Methods

### Study population

The rationale and design of the BEST study (Beijing Vascular Disease Patients Evaluation Study, Clinical Trials.gov Identifier: NCT02569268) have been described before [7]. Briefly, The BEST was a prospective cohort study of measuring all vascular parameters in patients with various related cardiovascular disease (CVD), including coronary artery disease (CAD), stroke, peripheral artery disease (PAD), hypertension, diabetes mellitus, dyslipidemia, as well as apparently healthy participants, and were consecutively recruited since 2010 from the vascular medicine center of Peking University Shougang Hospital in Beijing, China.

The present study was part of the BEST study and was aimed at systematic vascular evaluation, e.g. vascular ultrasound, arterial stiffness and blood examination. Subjects were enrolled if they were: (i) ≥18 years old; (ii) male or female; (iii) had a written informed consent. Exclusion criteria included: (i) deep venous thrombosis of lower extremities; (ii) known alcohol abuse; (iii) severe active liver and kidney disease; (iv) severe heart failure; (v) acute pulmonary embolism; (vi) abdominal aortic aneurysm.

The BEST study protocol conforms to the ethical guidelines of the 1975 Declaration of Helsinki and was approved by the Ethics committee of Peking University Shougang Hospital (the updated Ethics number IRBK-2017-017-01). Before enrollment, all study participants provided written informed consent.

### General clinical evaluation

The history of cardiovascular disease (CAD, Stroke, PAD, hypertension, diabetes, dyslipidemia), drugs (CVD drugs, hypoglycemic drugs, lipid-lowering drugs) and lifestyle (smoke and alcohol) were obtained by medical records. CVD drugs included angiotensin converting enzyme inhibitors (ACEI), Angiotensin Receptor Blocker (ARB), β-receptor blocker, calcium channel blockers and diuretics.

Systolic blood pressure (SBP), diastolic blood pressure (DBP) and heart rate (HR) were obtained by CF-PWV equipment. Body mass index was calculated as weight (kg) / height (m)^2^. Blood biochemical examination, including fasting blood glucose (FPG), total cholesterol (TC), triglyceride (TG), high density lipoprotein cholesterol (HDL-C), low density lipoprotein cholesterol (LDL-C), C-reactive protein (CRP), homocysteine (HCY), uric acid (UA), blood urea nitrogen (BUN) and creatinine (CR) was carried out by the clinical lab.

### Vascular ultrasound imaging to evaluate atherosclerotic plaque

**Fig. 1:**
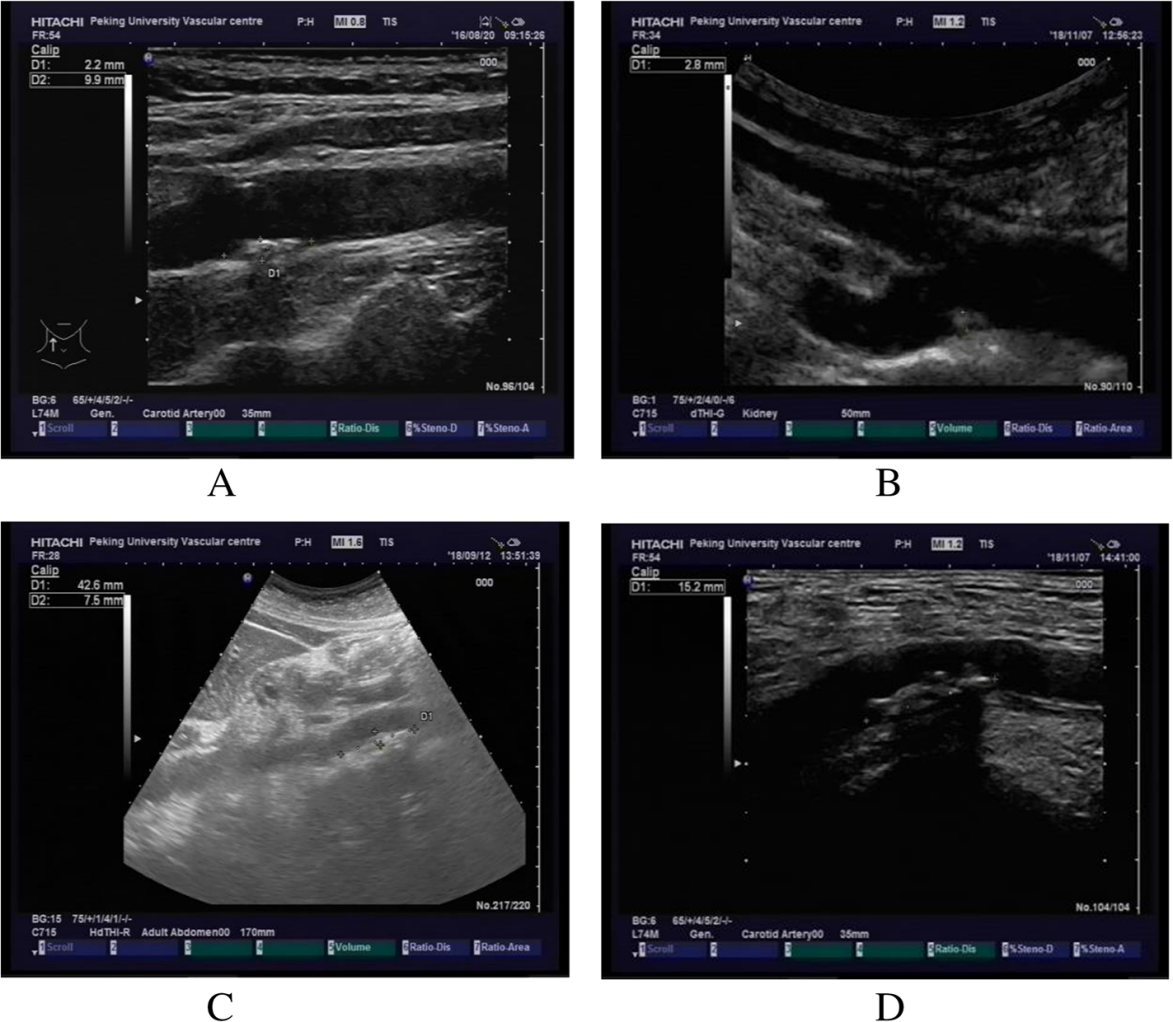
**a** Presence of plaque in carotid artery. **b** Presence of plaque in subclavian artery. **c** Presence of plaque in abdominal aorta. **d** Presence of plaque in femoral artery

The 2-dimensional vascular ultrasound (EUB 7500, Hitachi, Japan) were conducted by a linear array probe. The presence of atherosclerotic plaques was assessed through cross-sectional and longitudinal sweep of the right and left carotids and subclavian artery, the abdominal aorta, and the right and left lower extremities arteries. Plaque was defined as a focal protrusion into the arterial lumen of thickness ≥ 0.5 mm or ≥ 50% of the surrounding intima-media thickness or a diffuse thickness ≥ 1.5 mm measured between the media-adventitia and intima-lumen interfaces recommended by Manheim consensus [8]. The multi-site atherosclerotic plaques was defined according to the number of vascular sites affected (right or left carotid or subclavian artery = 1, abdominal aorta = 1, left or right lower extremities arteries = 1) (see Fig. 3a to d). Participants were grouped as 0 or 1 site affected (control group) and multi-site affected (multi-site atherosclerotic plaques group, 2 or 3 sites affected). Figure 1a-d showed the presence of plaque in carotid artery, subclavian artery, femoral artery and abdominal aorta.

### Detection of arteriosclerosis

CF-PWV was measured by an automated vascular equipment (Complior SP, Artech Medical, Pantin, France). The measurement was undertaken with the participant resting for 5 to 10 min in a supine position. CF-PWV was obtained from the right common carotid artery and the right femoral artery by inputting the pulse transit time and the distance between the carotid and femoral arteries. The foot-to-foot method was used for estimating the transit time, and the time delay (△t) was measured between the feet of the two waveforms. The foot of the wave was defined at the end of diastole, when the steep rise of the wavefront began. The transit time was the time of the foot wave traveling over a known distance. The distance (D) covered by the waves was assimilated to the surface distance between the two recording sites. CF-PWV was calculated as CF-PWV = D (meters)/ △t (seconds) [9]. CF-PWV was grouped as CF-PWV < 10 m/s, CF-PWV 10–12 m/s, CF-PWV > 12 m/s.

### Statistical analysis

Baseline characteristics were calculated using mean and standard deviation for continuous variables, count and proportions for categorical variables, and median and quartile for non-normally distributed variables. Differences between continuous variables and categorical variables were tested with unpaired t tests and χ2 tests, respectively. Non-normally distributed variables (triglyceride and C reactive protein) were tested with Man Whitney test. Multiple variables-adjusted associations between arteriosclerosis and multi-site atherosclerotic plaques were examined by use of logistic regression models. Age and sex-adjusted mean of predicted probability for multi-site atherosclerotic plaques (> = 2 sites) in each CF-PWV group were analyzed by covariance analysis. Statistical analyses were conducted with IBM SPSS Statistics Version 25.0. All analyses were two-sided, with a *P* value of 0.05 considered to indicate statistical significance.

## Results

### The general clinical characteristics of the study population, and the difference between control group (site <=1) and multi-site atherosclerotic plaques group (sites > = 2) (see Table 1)

The BEST cohort comprised 14,337 participants and 1178 in-hospital participants with complete data of systematic vascular ultrasound were enrolled into the study. General demographic characteristics and cardiovascular factors were summarized in Table 1. The average age of the participants was 67.4 years and 52% were male. The prevalence of CF-PWV > 12 m/s and multi-site atherosclerotic plaques were 40.2% and 74.4%. The most common cardiovascular factor was hypertension (74.1%), followed by dyslipidemia (73.9%), CAD (61.0%), stroke (39.8%), smoking (38.6%), diabetes (38.4%), PAD (30.8%) and alcohol (24.9%). Additionally, the proportion of participants with treatments were CVD drug (83.8%), antidiabetic drug (29.5%) and lipid-lowering drug (69.4%), respectively. Participants with multi-site atherosclerotic plaques (compared with site <=1) had higher level of age, SBP, CF-PWV, FPG, CRP, HCY, UA, BUN, CR, and had higher proportion of CF-PWV > 12 m/s, male, CAD, stroke, PAD, hypertension, diabetes, dyslipidemia, smoking, alcohol, drugs of CVD, antidiabetic drug and lipid-lowering drug, and had lower BMI, DBP, TC, HDL-C and LDL-C.

**Table 1:** Demographic Characteristics and Cardiovascular Factors

Variables	Total *N* = 1178	Site <=1 *N* = 301	*Site >=2* *N* = 877	*p* value
Age,year	67.40 ± 12.96	57.80 ± 11.11	70.70 ± 11.87	< 0.001
Male, n (%)	615(52.2)	117(38.9)	498(56.8)	< 0.001
BMI kg/m^2^	25.29 ± 3.68	25.92 ± 3.76	25.08 ± 3.63	.001
HR,beats/min	70.25 ± 13.89	69.52 ± 12.23	70.51 ± 14.41	.286
SBP, mmHg	143.30 ± 19.80	135.97 ± 17.59	145.82 ± 19.89	< 0.001
DBP, mmHg	84.41 ± 11.31	84.99 ± 11.31	84.21 ± 11.30	< 0.001
CF-PWV, m/s	11.66 ± 2.65	10.06 ± 1.76	12.22 ± 2.68	< 0.001
FPG, mmol/L	6.23 ± 2.28	5.94 ± 2.10	6.33 ± 2.33	.011
TC, mmol/L	4.34 ± 1.09	4.49 ± 1.04	4.29 ± 1.11	.006
TG, mmol/L	1.32(0.95–1.84)	1.35(1.00–1.99)	1.30(0.93–1.80)	.075
HDL-C, mmol/L	1.11 ± 0.28	1.15 ± 0.29	1.10 ± 0.27	.006
LDL-C, mmol/L	2.56 ± 0.78	2.66 ± 0.76	2.53 ± 0.78	.009
CRP, mg/L	1.73(0.79–4.54)	1.67(0.73–3.87)	1.79(0.82–4.83)	.024
HCY, umol/L	16.71 ± 7.72	15.00 ± 6.86	17.30 ± 7.91	< 0.001
UA, umol/L	331.49 ± 94.37	316.96 ± 88.37	336.48 ± 95.88	.002
BUN, mmol/L	5.55 ± 1.90	5.04 ± 1.49	5.72 ± 1.99	< 0.001
CR, umol/L	74.09 ± 24.69	66.07 ± 23.21	76.86 ± 24.60	< 0.001
History of Diseases				
CAD, n (%)	719(61.0)	137(45.5)	582(66.4)	< 0.001
Stroke, n (%)	464(39.8)	58(19.4)	406(46.8)	< 0.001
PAD, n (%)	359(30.8)	20(6.7)	339(39.1)	< 0.001
Hypertension, n (%)	873(74.1)	186(61.8)	687(78.3)	< 0.001
Diabetes, n (%)	452(38.4)	75(24.9)	377(43.1)	< 0.001
Dyslipidemia, n (%)	870(73.9)	200(66.4)	670(76.5)	.001
Without any above diseases	40(3.4%)	27(9.0%)	13(1.5%)	< 0.001
Drugs				
CVD drugs, n (%)	987 (83.8)	209(69.4)	778(88.7)	< 0.001
Antidiabetic drugs, n (%)	347(29.5)	58(19.3)	289(33.0)	< 0.001
Lipid-lowering drugs, n (%)	817(69.4)	171(56.8)	646(73.7)	< 0.001
Life style				
History of smoke, n (%)	452(38.6)	82(27.3)	370(42.4)	< 0.001
History of alcohol, n (%)	292(24.9)	62(20.7)	230(26.4)	.048
CF-PWV				< 0.001
CF-PWV (< 10 m/s), n (%)	330(29.2)	158(54.3)	172(20.5)	
CF-PWV (10–12 m/s), n (%)	345(30.6)	89(30.6)	256(30.5)	
CF-PWV (> 12 m/s), n (%)	454(40.2)	44(15.1)	410(48.9)	< 0.001

Data are expressed as mean ± SD, % (n), or median (interquartile range) when appropriate. *P* values are derived from independent t tests for continuous variables and χ2 for categorical variables. Abbreviations see List of Abbreviations

### Association between multi-site atherosclerotic plaques and CF-PWV as well as other factors, results from multivariable logistic regressions (see Table 2)

The results of multivariable logistic regressions were showed in Table 2, with age, male, BMI, HR, SBP, DBP, CF-PWV, FPG, TC, TG, HDL-C, LDL-C, CRP, HCY, UA, BUN, CR, diseases history (CAD, Stroke, PAD, hypertension, diabetes, dyslipidemia), drugs (CVD drugs, hypoglycemic drugs, lipid-lowering drugs) and life style (smoke, alcohol) as independent variables. Eventually, CF-PWV > 12 m/s (odds ratio, OR 2.249, 95% Confidence Interval, 95% CI 1.379 to 3.668, *p* = 0.001), age (OR 1.085, 95% CI 1.065 to 1.105, *p* < 0.001), male sex (OR 1.842, 95% CI 1.169 to 2.901, *p* = 0.008), stroke (OR 1.638, 95% CI 1.097 to 2.445, *p* = 0.016), PAD (OR 3.882, 95% CI 2.158 to 6.982, *p* < 0.001), dyslipidemia (OR 1.894, 95% CI 1.274 to 2.815, *p* = 0.002), hypoglycemic drugs (OR 1.555, 95% CI 1.034 to 2.339, *p* = 0.034), and smoking (OR 2.485, 95% CI 1.522 to 4.057, *p* < 0.001) were included into the model. In participants with CF-PWV > 12 m/s, the odds ratio (OR) for multi-site atherosclerotic plaques was 2.249 comparing with CF-PWV < 10 m/s, independently of age, sex, and other CVD factors. In addition, patients with PAD showed the highest association (OR 3.882, 95% CI 2.158 to 6.982, *p* < 0.001) with multi-site atherosclerotic plaques, followed by smoking (OR 2.485, 95% CI 1.522 to 4.057, p < 0.001), CF-PWV > 12 m/s (OR 2.249, 95% CI 1.379 to 3.668, *p* = 0.001), dyslipidemia (OR 1.894, 95% CI 1.274 to 2.815, *p* = 0.002), male sex (OR 1.842, 95% CI 1.169 to 2.901, *p* = 0.008), stroke (OR 1.638, 95% CI 1.097 to 2.445, *p* = 0.016), hypoglycemic drugs (OR 1.555, 95% CI 1.034 to 2.339, *p* = 0.034) and age (OR 1.085, 95% CI 1.065 to 1.105, p < 0.001).

**Table 2:** Relationship with multiple plaque territories of CF-PWV and other factors, results from multivariable Logistic regressions

	MAP (Site <=1 as reference)			
Variables	B	OR	95% CI	P
CF-PWV				
CF-PWV (< 10 m/s)	Ref.	–	–	–
CF-PWV (10–12 m/s)	0.200	1.222	0.811 to 1.841	.338
CF-PWV (> 12 m/s)	0.810	2.249	1.379 to 3.668	.001
Age,year	0.081	1.085	1.065 to 1.105	.000
Sex				
Female	Ref.			
Male	0.611	1.842	1.169 to 2.901	.008
Stroke	0.493	1.638	1.097 to 2.445	.016
PAD	1.356	3.882	2.158 to 6.982	.000
Dyslipidemia	0.639	1.894	1.274 to 2.815	.002
Hypoglycemic agents	0.442	1.555	1.034 to 2.339	.034
History of Smoke	0.910	2.485	1.522 to 4.057	.000
CRP, mg/L	0.014	1.015	1.000 to 1.029	.051

Abbreviations as in List of Abbreviations. Adjusted: Age, Male, BMI, HR, SBP, DBP, CF-PWV, FPG, TC, TG, HDL-C, LDL-C, CRP, HCY, UA, BUN, CR, Diseases history (CAD, Stroke, PAD, hypertension, diabetes, dyslipidemia), Drugs (CVD drugs, Hypoglycemic drugs, Lipid-lowering drugs), Life style (Smoke, Alcohol)

### Prevalence and vascular distribution of atherosclerotic plaque, in male and female (see Fig. 2a-d)

The proportion of plaque formation in 0, 1, 2 and 3 sites were 6.6%, 18.9%, 31.2% and 43.2% respectively. Most participants (74%) had multi-site atherosclerotic plaques. Plaques formation were detected in 93.4% of participants (6.5% without any plaque, 90.1% in the carotids, 70.5% in the lower extremities arteries, and 50.5% in the abdominal aorta). In addition, 31.2% and 43.2% of the participants were with 2 and 3 sites affected. In male, atherosclerotic plaque formation in 3 sites was more prevalent (48.9% versus 36.9% in female, *p* < 0.001), and also more prevalent across all single vascular site in carotid (92.7% versus 87.2% in female, *p* < 0.05) or lower extremities artery (77.2% versus 63.1% in female, p < 0.001) or abdominal aorta (55.1% versus 45.5% in female, p < 0.05).

**Fig. 2:**
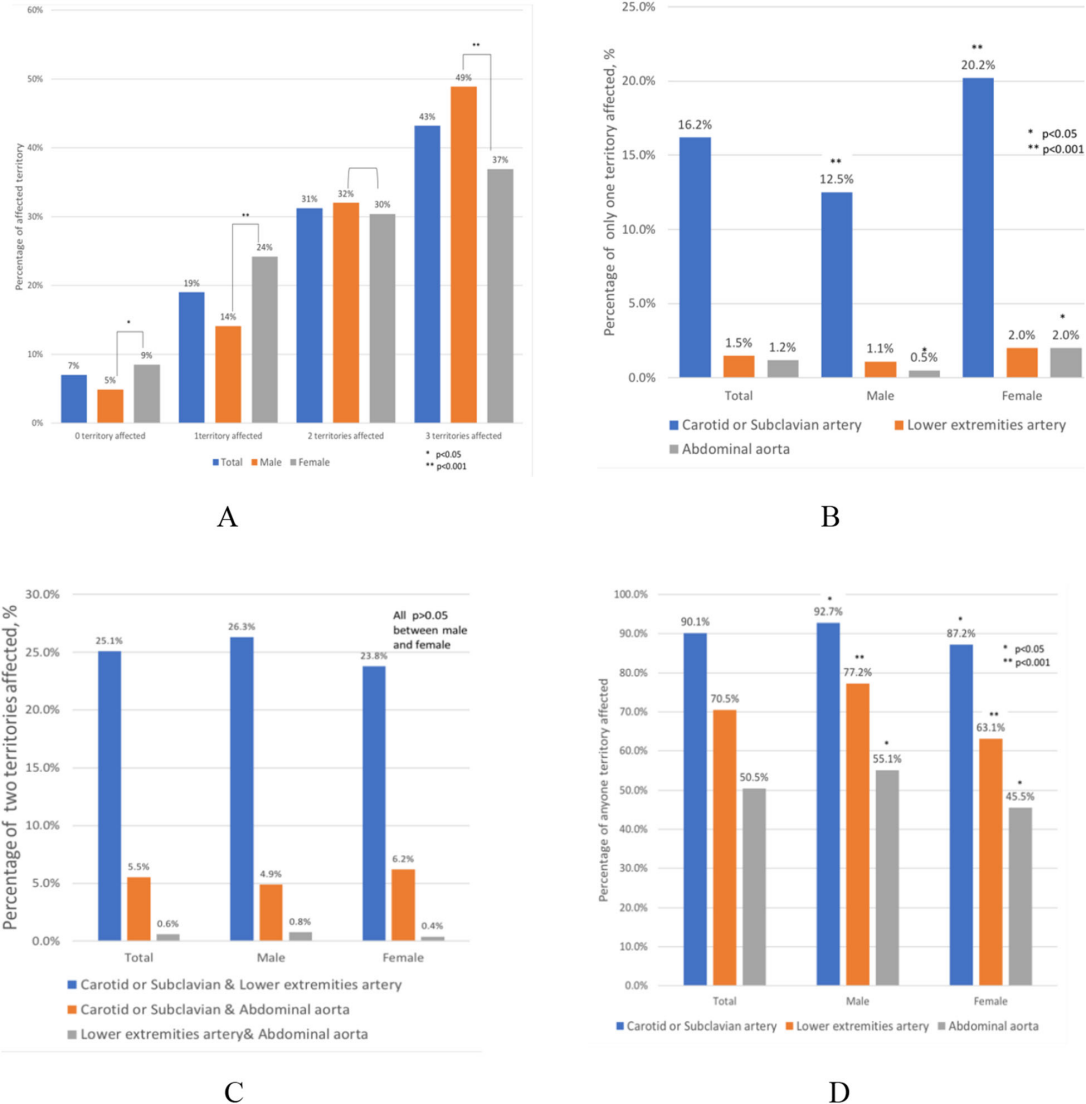
**a** to **d** Prevalence and Distribution of Atherosclerotic Plaque in different vascular sites (in each single or combined vascular site) by ultrasonography, comparing male with female. Vascular sites examined were the right and left carotids and subclavian artery, the abdominal aorta, and the right and left lower extremities arteries (presence of plaque). **a** The distribution of the number of plaque site detected by vascular ultrasound, including presence of plaque in 0,1,2,3 of carotid artery, abdominal aorta, femoral artery, comparing male with female. **b** The distribution of plaque site in only one of carotid artery, femoral artery and abdominal aorta detected by vascular ultrasound, comparing male with female. **c** The distribution of plaque site in two of carotid artery, femoral artery and abdominal aorta detected by vascular ultrasound, comparing male with female. **d** The individual distribution of anyone territory plaque in carotid artery, femoral artery and abdominal aorta detected by vascular ultrasound, comparing male with female

### Age and sex-adjusted mean of predicted probability of multi-site atherosclerotic plaques (> = 2 sites) in each CF-PWV group, analyzed by age and sex-adjusted covariance analysis (see Fig. 3)

Participants with CF-PWV < 10 m/s, CF-PWV 10–12 cm/s and CF-PWV > 12 m/s corresponded to an age and sex-adjusted mean 64%, 73% and 82% probability of multi-site atherosclerotic plaques, respectively.

**Fig. 3:**
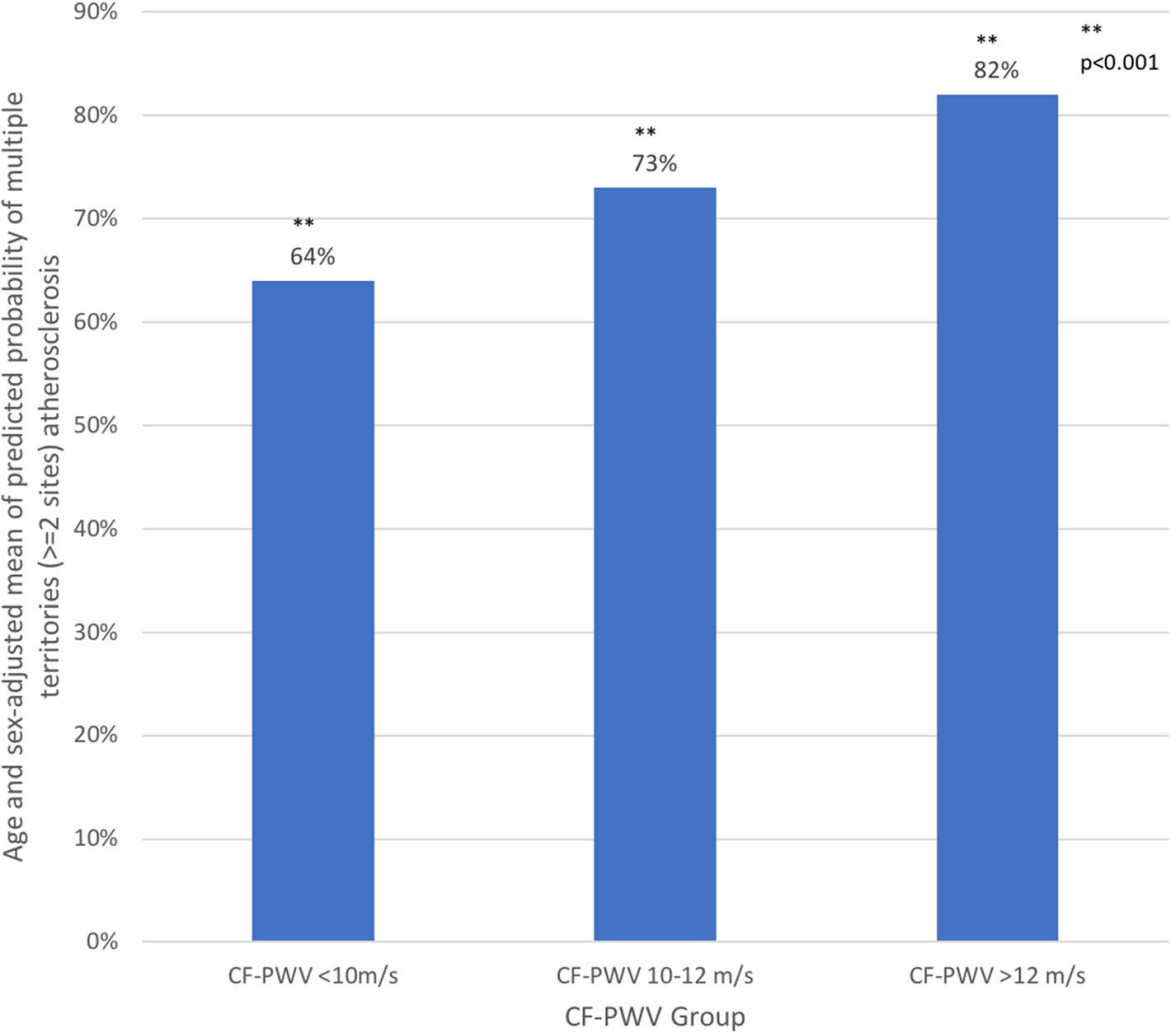
Age and sex-adjusted mean of predicted probability of multi-site atherosclerotic plaques (> = 2 sites) in each CF-PWV group, analyzed by age and sex-adjusted covariance analysis

## Discussion

The study cohort is designed to explore the cross-sectional relationship between multiple atherosclerosis and arterial stiffness, which is of high clinical value for the comprehensive evaluation of systematic atherosclerotic burden by serial plaques assessment as it has additive clinical value to classical (traditional) atherosclerosis risk factors [10]. In addition, study found that maintained carotid intima-media thickness (CIMT) regression is associated with 68 to 75% reduction in the risk of a cardiovascular event. However, a long-term maintained CIMT regression is achieved in one-fourth of patients with either CAD or PAD [10].

The results of the present study showed that the prevalence of CF-PWV > 12 m/s and multi-site atherosclerotic plaques (> = 2 sites) were 40.2% and 74.4%, respectively. Multi-site atherosclerotic plaques were highly prevalent in this in-hospital cohort, and male showed a higher prevalence than female. Participants with multi-site atherosclerotic plaques had higher level of cardiovascular factors and higher arteriosclerosis. In addition, CF-PWV > 12 m/s corresponded to an age and sex-adjusted mean 82% probability of multi-site atherosclerotic plaques. Most important of all, higher arteriosclerosis was independently associated with multi-site atherosclerotic plaques, which suggested the added value of arteriosclerosis for the identification of multi-site atherosclerotic plaques.

### The prevalence of multi-site atherosclerotic plaques

A study used whole-body magnetic resonance angiography to assessed the prevalence and distribution of atherosclerosis in 1528 participants with 10-year risk of cardiovascular disease less than 20%, the results showed 49.4% participants had at least one stenotic vessel, and 27.0% participants had multiple stenotic vessels, and pointed out although disease prevalence was low on a per-vessel level, vascular disease was common on a per-participant level, even in a low- to intermediate- risk cohort [11]. The progression of early subclinical atherosclerosis (PESA) study including 4184 asymptomatic participants 40 to 54 years of age was designed to evaluate the systemic extent of atherosclerosis in the carotid, abdominal aortic, and iliofemoral territories by ultrasound. They found subclinical atherosclerosis was present in 63% of participants, and 83% of participants at high risk had atherosclerosis, with 66% classified as intermediate or generalized [12]. Inspired by the previous study and as data supplement for current research from China, the present study including in-hospital participants (mean age 67.40 years, 61.0% CAD, 39.8% stroke, 30.8% PAD, 74.1% hypertension, 38.4% diabetes, 73.9% dyslipidemia, 3.4% without any above diseases), only about 7% were free of atherosclerosis in any of the three vascular sites, and the prevalence of multi-site atherosclerotic plaques was 74.4% which was similar to other studies. To further evaluate the prevalence in different vessel bed, we found in the studied population from China, with carotid plaque was the most common, which was different from the PESA in which presence of ilio-femoral artery was the most common [12, 13]. The main reasons are that the study population (asymptomatic vs high-risk participants) and different high incidence diseases (coronary heart disease vs stoke) vary from other country to China. More research tips showed, among 1812 subjects (49% female, 21% black, 14% Chinese, and 25% Hispanic), the presence of multi-site atherosclerosis (≥ 3 of the abdominal aorta calcium, coronary artery calcium, ankle brachial index, carotid intimal medial thickness) ranged from 20% in women and 30% in men, and was highest in Caucasians (28%) and lowest in Chinese (16%) [14]. As for the presence of plaque in any vessel, previous studies have also assessed. For carotid artery, the prevalence rate varied from 78 to 87% [15, 16]. In addition, in the present study, male showed a higher prevalence of multi-site atherosclerosis than female which was consistent with the previous data [6, 12, 13].

### Association between multi-site atherosclerotic plaques and arteriosclerosis, as well as other factors

Studies showed that traditional risk factors including aging, hypertension, current smoking, diabetes and the level of HDL-C were associated with subclinical carotid atherosclerosis and plaque burden [6, 15, 17]. In our study from in-hospital population, we also found CF-PWV, age, sex, stroke, PAD, dyslipidemia, hypoglycemic agents and smoke were independently associated with multi-site atherosclerosis. As an important finding, CF-PWV > 12 m/s was an independent factor related to multi-site atherosclerotic plaques compared with CF-PWV < 10 m/s, which indicated the potential impact of vascular function on vascular structure. As for the important influence of combine vascular lesions on cardiovascular events had been explored by various studies [4, 5, 18, 19]. A study assessed the relation of Framingham risk score (FRS) to subclinical atherosclerosis evaluated across three arterial sites, by measuring subclinical atherosclerosis in the coronary arteries and aorta with the presence of calcium and in the common carotid artery by intima-media thickness in 498 healthy subjects. The results showed the FRS were significantly independently associated with these subclinical atherosclerosis measures of the three arterial sites and increased with the number of arterial sites with atherosclerosis [19]. The BioImage Study directly quantifying atherosclerosis in different vascular beds performed in a single cohort indicated major adverse cardiac events rates increased simultaneously with higher levels of both carotid plaque burden and coronary artery calcification [4]. A study in Male Marathon Runners found the prevalence of carotid and peripheral atherosclerosis in 100 marathon runners was high and was related to cardiovascular risk factors and the coronary atherosclerotic burden [18]. Furthermore, CF-PWV as the gold standard for arterial stiffness, has been confirmed the predicting value for future cardiovascular disease and recommended by various guidelines [1–3]. Therefore, the present cross-sectional study showed some indication for the relationship between arteriosclerosis and multi-site atherosclerosis, which maybe as an earlier marker for the evaluation of multi-site atherosclerosis. Our follow-up data will further confirm the value of arteriosclerosis as an earlier marker.

## Conclusions

In conclusion, our study with a large clinic sample from Beijing, China, found the significant associations between arteriosclerosis and multi-site atherosclerotic plaques. In this high-risk cohort, multi-site atherosclerotic plaques were highly prevalent and male showed a higher prevalence than female. Higher arteriosclerosis was independently associated with multi-site atherosclerotic plaques suggesting the adding value of arteriosclerosis for the identification of multi-site atherosclerosis. Further studies with pre-designed and prospective data collection are warranted to confirm our findings.

### Limitations of this study

1. The present study was a cross-sectional analysis of the BEST cohort at baseline and cannot provide the evidence of a causal relationship, although we made the assumptions that these vascular-related factors may cause atherosclerosis.
2. The participants were from a single center and the BEST sample was consisting of high-risk participants with established cardiovascular disease, which may limit the wide generalizability.
3. Although the prevalence of diseases might not be universally representative given the specific characteristics of our participants, the observed associations between arteriosclerosis and multi-site atherosclerosis could be extrapolated to other cohorts.
4. Anyway, the present analysis set the basis for the understanding of the association between vascular function and vascular structure.

## Data Availability

The datasets analyzed during the current study are available from the corresponding author on reasonable request.

## Abbreviations

MAP: Multi-site atherosclerotic plaques
BMI: Body mass index
HR: Heart rate
SBP: Systolic blood pressure
DBP: Diastolic blood pressure
CF-PWV: Carotid femoral artery pulse wave velocity
FPG: Fasting plasma glucose
TC: Total cholesterol
TG: Triglyceride
HDL-C: high-density lipoprotein cholesterol
LDL-C: low-density lipoprotein cholesterol
CRP: C reactive protein
HCY: Homocysteine
UA: Uric acid
BUN: Blood urea nitrogen
CR: creatinine
CAD: Coronary artery disease
PAD: Peripheral artery disease
CVD: Cardiovascular disease
CVD drugs: One of angiotensin converting enzyme inhibitors, Angiotensin Receptor Blocker, β-receptor blocker, calcium channel blockers and diuretics.
